# Depression is associated with enhanced aversive Pavlovian control over instrumental behaviour

**DOI:** 10.1038/s41598-018-30828-5

**Published:** 2018-08-22

**Authors:** C. L. Nord, R. P. Lawson, Q. J. M. Huys, S. Pilling, J. P. Roiser

**Affiliations:** 1 Institute of Cognitive Neuroscience, University College London, 17 Queen Square, WC1N 3AZ London, UK; 2 Department of Psychiatry, University of Cambridge, Addenbrookes Hospital, Level E4, Box 189, Hills Road, CB2 0QQ Cambridge, UK; 3 Wellcome Trust Centre for Neuroimaging, University College London, 12 Queen Square, WC1N 3BG London, UK; 4 Department of Psychology, University of Cambridge, Downing Street, CB2 3EB Cambridge, UK; 5 Translational Neuromodeling Unit, Institute for Biomedical Engineering, ETH Zürich and University of Zürich, Wilfriedstrasse 6, 8032 Zürich, Switzerland; 6 Department of Psychiatry, Psychotherapy and Psychosomatics, Hospital of Psychiatry, University of Zurich, Lenggstasse 31, 8032 Zürich, Switzerland; 7 Department of Clinical, Educational and Health Psychology, University College London, Gower Street, WC1E 6BT London, UK

## Abstract

The dynamic modulation of instrumental behaviour by conditioned Pavlovian cues is an important process in decision-making. Patients with major depressive disorder (MDD) are known to exhibit mood-congruent biases in information processing, which may occur due to Pavlovian influences, but this hypothesis has never been tested directly in an unmedicated sample. To address this we tested unmedicated MDD patients and healthy volunteers on a computerized Pavlovian-Instrumental Transfer (PIT) task designed to separately examine instrumental approach and withdrawal actions in the context of Pavlovian appetitive and aversive cues. This design allowed us to directly measure the degree to which Pavlovian cues influence instrumental responding. Depressed patients were profoundly influenced by aversive Pavlovian stimuli, to a significantly greater degree than healthy volunteers. This was the case for instrumental behaviour both in the approach condition (in which aversive Pavlovian cues inhibited ‘go’ responses), and in the withdrawal condition (in which aversive Pavlovian cues facilitated ‘go’ responses). Exaggerated aversive PIT provides a potential cognitive mechanism for biased emotion processing in major depression. This finding also has wider significance for the understanding of disrupted motivational processing in neuropsychiatric disorders.

## Introduction

Human behaviour automatically adapts to suit rapidly-changing situational demands^1^. Cues predicting rewarding or aversive outcomes can facilitate adaptive behaviour; for example, water cues guide us to find and drink when thirsty^2^. A disruption in the mechanisms underlying adaptation to positively- or negatively-valenced environmental cues is thought to play a key role in many psychiatric symptoms^3,4^. As such, environmental cues do not always trigger adaptive behaviours: the same mechanism guiding us to seek water is thought to mediate the facility with which drug cues provoke drug-seeking in patients recovered from substance dependence^5^.

Even irrelevant environmental cues can profoundly alter goal-seeking behaviour. Pavlovian-instrumental transfer (PIT) is defined as the ability of passively-conditioned (Pavlovian) cues to automatically invigorate (and in some cases suppress) ongoing goal-directed instrumental behaviour. Importantly, Pavlovian stimuli exert influence over instrumental performance despite no formal association between Pavlovian and instrumental contingencies^6^.

PIT arises from the interaction between two associative learning processes. The first, a Pavlovian incentive learning system passively associates the sensory aspects of an event with its motivational outcomes^7^. Environmental cues accrue value (appetitive or aversive) through this passive Pavlovian process independent of any response made by the subject. These passively-conditioned cues can interact with the second associative learning process, an instrumental learning system. In contrast to the first system, the value of instrumental outcomes is contingent on the specific actions performed. For example, a rat might encode the association between a lever press and food access (instrumental) and separately learn passively that a flash of light predicts shock delivery, irrespective of any lever press (Pavlovian). Crucially, these processes are dissociable^8^. Even without any direct association between passively-learned Pavlovian associations and instrumental contingencies, Pavlovian cues can unconsciously invigorate or suppress concurrent instrumental responding, in both humans^6^ and animals^7^.

Animal studies have contributed a rich understanding of the neurobiology of Pavlovian influences on instrumental behaviour^9–11^. This work has largely examined PIT in the context of appetitive stimuli, linking it specifically to dopamine transmission: dopamine D1 and D2 receptors in the nucleus accumbens (NAcc) core and shell mediate the general activating effects of appetitive Pavlovian stimuli on instrumental behaviour^12^, while inactivation of the ventral tegmental area (VTA) decreases appetitive PIT^13^. Recent studies have employed comparable PIT paradigms in humans^6,14–16^, suggesting that the circuits driving this process are similar in humans and animals. These studies have also begun to explore in more detail the effects of aversive Pavlovian stimuli on instrumental behaviour, implicating the ventromedial prefrontal cortex and caudate in behavioural suppression^14^, as well as the serotonergic system, which may play a larger role in aversive than appetitive PIT^15^.

A disruption in the mechanisms that mediate PIT could drive various aspects of psychiatric symptomatology. Major depressive disorder (MDD) is characterised by mood-congruent biases in information processing: depressed patients have facilitated recall of negative over positive material^17^, and in a seminal study, were found to show an exaggerated influence of explicit negative feedback on performance^18^. The latter result is sometimes described as a ‘catastrophic response to perceived failure’^18^, and was later confirmed in work specifically designed to assess response to negative feedback^19^.

These findings support the broader theoretical proposal that depression involves maladaptive (hyper-sensitive) responses to negative stimuli, as well as an under-responsivity to positive material^3^. This exaggerated influence of negative stimuli likely warps patients’ expectations and interpretations of their environment, making important contributions to the cognitive processes that drive depressive symptomology^20^. A major unanswered question is precisely how negative biases might influence behaviour.

If behaviour in depression is disproportionately guided by negative processing, we might expect an enhancement of aversive PIT (representing a greater ability of aversive Pavlovian stimuli to automatically increase instrumental withdrawal behaviour), and a suppression of appetitive PIT (representing a lesser ability of appetitive Pavlovian stimuli to invigorate instrumental approach behaviour). If this is the case, this disrupted PIT could explain how passively-reinforced cues could encourage avoidance and discourage approach behaviour in depression.

A previous study found that Pavlovian stimuli did not exert action-specific effects on instrumental behaviours in depressed patients, but that greater action-specificity in patients was associated with recovery from depression^16^. However, this study did not exclude patients who were currently taking antidepressant medication, a critical issue as most antidepressants alter serotonergic processing, which could significantly affect PIT, particularly in the aversive domain^15^, due to serotonin’s role in affective processing^21^. While the authors ran an analysis covarying for medication status, the study was likely insufficiently powered for this analysis, and therefore could not address this issue.

The rationale for the study was to investigate PIT in depression without the potentially confounding effects of current medication. To do so, we recruited only unmedicated patients (minimum four weeks off-medication), an approach employed in a number of studies to investigate abnormalities in cognitive processing that are an effect of psychiatric illness itself (for example, abnormalities in decision making in bipolar depression have been found in medicated as well as unmedicated patients^22^) from those attributable to medication (for example, latent inhibition in schizophrenia has been reported to be disrupted only in individuals exposed to antipsychotics^23^).

To test this, we administered a computerized PIT paradigm to unmedicated currently depressed adults and a matched group of healthy volunteers. The PIT task we employed dissociated approach and withdrawal behaviour from behavioural activation and inhibition (i.e. go or no-go), which allowed us to examine whether Pavlovian stimuli influence the value of instrumental behaviour, or simply promote a go (or no-go) response^24^, and whether this interaction is altered in depression. Based on the above hypothesis, we predicted that unmedicated depressed patients would show an excessive influence of aversive Pavlovian cues, and a diminished influence of appetitive Pavlovian cues, although this was contrary to the previous study’s findings^16^.

## Methods

### Participants

Twenty-six patients with major depressive disorder (MDD) and 28 healthy volunteers group-matched for age, gender, and intelligence quotient (IQ: assessed by the Wechsler Test of Adult Reading^25^) were recruited to take part in the study (demographic and clinical data are displayed in Table 1). Patients were recruited via referral from the Camden and Islington NHS Foundation Trust, or following completion of an advertised online screening questionnaire. Healthy volunteers were recruited via the University College London Psychology department online subject pool. All participants were screened for current or previous neuropsychiatric disorder using the Mini International Neuropsychiatric Interview (MINI, version 5.0.0^26^), and depressive symptoms were assessed with the Hamilton Depression Rating Scale (HAM-D)^27^. No participants were taking psychiatric medication at the time of the study, or had taken any recreational drugs in the six weeks leading up to the study. Current antidepressant medication use was assessed through participant report.

**Table 1:** Descriptive statistics for patient (MDD) and control groups.

	Controls (n = 28)	MDD (n = 26)	Test statistic	Degrees of freedom	Significance
% female	46.43	38.46	*X*^2^ = 0.350	1	0.56
Age, mean (s.d.)	26.79 (8.48)	27.96 (8.75)	*t* = 0.50	52	0.62
Predicted IQ, mean (s.d.)	111.22 (4.91)	110.85 (6.02)	*t* = 0.25	51	0.80
HAM-D, mean (s.d.)	1.57 (1.48)	18.84 (3.92)	*t* = 21.37	31.13	<0.001
BDI, mean (s.d.)	3.43 (4.03)	23.96 (9.29)	*t* = 10.40	33.55	<0.001
SHAPS, mean (s.d.)	0.89 (1.74)	5.62 (3.40)	*t* = 6.34	36.97	<0.001
No. episodes MDD, mean (s.d.)	NA	9.74 (9.41)	NA	NA	NA
Age of 1st episode, mean (s.d.)	NA	18.46 (5.57)	NA	NA	NA
% previous antidepressant use	NA	42.31	NA	NA	NA
% attempted suicide	NA	34.62	NA	NA	NA

Predicted IQ computed from Weschler Test of Adult Reading. HAM-D (Hamilton Depression Rating scale); BDI (Beck Depression Inventory); SHAPS (Snaith-Hamilton Pleasure Scale).

Exclusion criteria for healthy controls were any past or present psychiatric or neurological disorder. All patients met criteria for MDD on the MINI and were currently in a depressive episode. Exclusion criteria for patients were: recent psychotropic medication use (within four weeks of participation, or eight for fluoxetine); history of manic or hypomanic episodes; history of psychosis; or history of substance abuse/dependence (unless confined to a depressive episode). Anxiety disorders did not constitute an exclusion criterion for MDD participants; 62% of the patient sample additionally met criteria for an anxiety disorder at the time of the study.

Both patients and controls completed a self-rating questionnaire measure of depression (Beck Depression Inventory (BDI)^28^) and anhedonia (Snaith-Hamilton Pleasure Scale (SHAPS)^29^): see Table 1.

The study was approved by the London Queen Square NHS Ethics Committee, and all methods were performed in accordance with their relevant guidelines and regulations. All participants gave written informed consent for the study and were informed that their payment was performance-dependent (though all were compensated the same amount (£10) upon completion of the experiment).

### PIT task

**Figure 1:**
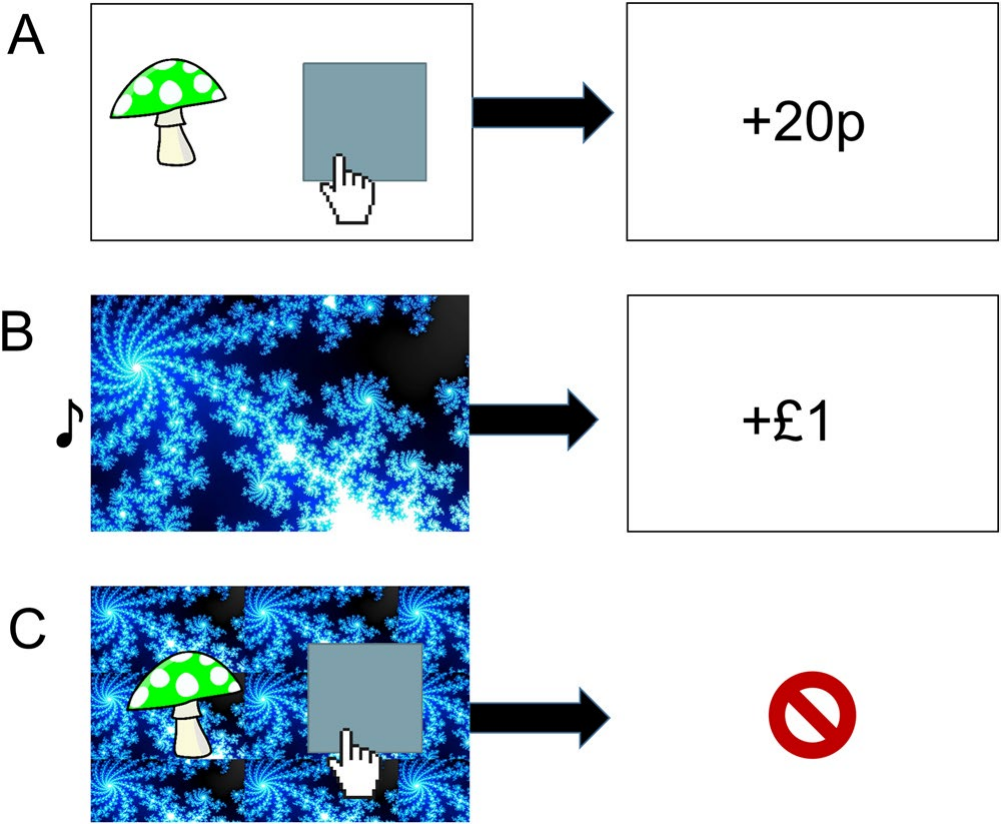
Task Description. (**A**) Instrumental learning (Stage 1). Participants clicked in the box to collect a win-associated mushroom in the approach block, or to reject a loss-associated mushroom in the withdrawal block. (**B**) Pavlovian training (Stage 2). Participants passively observed different fractal images followed by associated gains and losses. Each fractal was associated with a uniquely pitched tone that played simultaneously (**C**). Pavlovian-instrumental transfer (Stage 3). Instructions were identical to Stage 1 but no reinforcement was given. The Pavlovian fractal images appeared tiled in the background.

Participants were administered a computerized PIT task written using Psychtoolbox in Matlab (http://psychtoolbox.org)^24^. The task comprised three stages: 1) Participants learned the instrumental contingencies of various actions; 2) They passively observed Pavlovian stimuli paired with different outcomes; 3) They performed the instrumental task again, but with Pavlovian stimuli presented in the background (see Fig. 1).

#### Stage 1

Participants first performed an instrumental training task (see Fig. 1A), with the aim of maximizing their earnings. There were two conditions in this task. In the approach condition, participants were instructed to actively ‘collect’ stimuli (coloured mushrooms) associated with monetary wins by clicking the mouse, while not making any response in order to avoid those associated with losses. In the withdrawal condition, participants were instructed to actively ‘discard’ stimuli associated with losses by clicking the mouse, while not making any response in order to collect those associated with gains. The approach and withdrawal conditions were completed in different blocks, each comprising 120 trials.

This instrumental task required participants to learn the probabilistic values of six different cartoon mushrooms, half of which resulted in a monetary gain 80% of the time, and a loss 20% of the time; the other half resulted in a monetary loss 80% of the time, and a gain 20% of the time. After each choice, feedback of +£0.20 or −£0.20 was displayed on the screen either immediately after a ‘go’ action (mouse click) or 1.5 seconds after the stimulus was displayed if no response (‘no-go’) was made. The order of approach/withdrawal blocks was counterbalanced across participants, as were the associations between the mushroom stimuli and outcomes.

#### Stage 2

Participants next passively observed as one of five different fractal images were displayed on the screen in a random order, each paired with a uniquely pitched tone (Fig. 1B). One second after stimulus presentation, each image was followed by one of five outcomes: a large win ( £1), a small win (+£0.1), no win (£0), a small loss (−£0.1), or a large loss (−£1). Every fifth trial, the participants were required to choose the better of two of the fractal images. These Pavlovian choice trials served as a measure of the passive Pavlovian conditioning. Each outcome amount (£1, £0.1, £0, £-0.1, and −£1, corresponds to a Pavlovian stimulus valence (large win, small win, neutral, small loss, and large loss, respectively). This stage comprised 90 trials.

#### Stage 3

Finally, in the PIT stage, participants were again instructed to perform the instrumental mushroom gathering task (Fig. 1C). However, in this stage the instrumental stimuli were superimposed on a background of one of the Pavlovian fractal images, and performed in extinction (i.e. without any reinforcing outcomes for either Pavlovian or instrumental stimuli). There were 200 trials in each of the approach and withdrawal blocks in this stage, each lasting until the subject performed a ‘go’ action, or after 1.5 seconds if no response was made (with no inter-trial interval). Each trial randomly sampled a Pavlovian image and instrumental mushroom stimulus to randomize cue combinations across participants and control for any stimulus-specific effects.

We measured performance in Stage 1 (initial instrumental stage: percent correct go and no-go responses, in the approach and withdrawal conditions separately), Stage 2 (Pavlovian training stage: percent correct choices), and in Stage 3 (PIT stage: probability of making a go response for each Pavlovian background, in the approach and withdrawal conditions separately).

The following image used in Fig. 1A,C is reproduced under the terms of a Creative Commons CCO 1.0 Universal license (https://creativecommons.org/publicdomain/zero/1.0/deed.en) and has been adapted from its original form. The link to the original image is: (https://pixabay.com/en/mushroom-fly-agaric-green-fairytale-311045/). The following image used in Fig. 1A,C is reproduced under the terms of a Creative Commons 2.5 Generic license (https://creativecommons.org/licenses/by/2.5/deed.en) and has been adapted from its original form (Original Author: Lordalpha1; title: A vector hand cursor and cursor). The link to the original image is: https://commons.wikimedia.org/wiki/File:Mouse-cursor-hand-pointer.svg. The following image used in Fig. 1B,C was reproduced under the terms of a Creative Commons Attribution-ShareAlike 3.0 Unported License (https://creativecommons.org/licenses/by-sa/3.0/deed.en) and has been adapted from its original form (Original Author: Kh627; title: Mandelbrot fractal). The link to the original image is: https://commons.wikimedia.org/wiki/File:Mandelbrot_fractal_rendered_in_Paint.NET.jpg. The musical note (Fig. 1B) is a Unicode Character, ‘Eighth Note’ (U + 266 A) (The Unicode Standard, Version 1.1.0, (Mountain View, CA: The Unicode Consortium, 1993).

### Analysis

Analysis was conducted in IBM SPSS Statistics for Windows, Version 22.0 (Armonk, NY: IBM Corp). The distributions of all measures of interest were checked prior to analysis and none deviated substantially from Gaussian assumptions. We first analysed the effects of instrumental condition (approach/withdrawal) and action (go/no-go) on accuracy in the instrumental learning stage (Stage 1), using a repeated-measures analysis of variance (ANOVA), including group as a between-subjects factor. We then tested whether accuracy on the Pavlovian choice trials (in Stage 2) differed between groups with an independent samples t-test. To check that participants retained the associations from Stage 1 in Stage 3, we repeated the same repeated measures ANOVA as for Stage 1. Finally, to test our key hypothesis and specifically examine the PIT effect in Stage 3, we conducted a repeated-measures ANOVA on the probability of making a ‘go’ response, with instrumental condition (approach/withdrawal) and Pavlovian stimulus valence (large win/small win/neutral/small loss/large loss) as within-subjects factors, again including group as a between-subjects factor. Pearson’s *r* correlations were used to assess the relationship between depressive symptoms (HAM-D scores) and summary measures describing the aversive and appetitive PIT effect in each participant. In all analyses, we considered *p* < 0.05 as significant.

### Power analysis

We computed that 52 subjects (26 per group) were needed to observe a large effect size of d = 0.8 with 80% power (statistical test: difference between two independent means (two groups) calculated using G*Power 3.1.9.2)). At the time the study was planned, no previous study had reported a difference between any patient group and controls on PIT. We considered an effect size of 0.8 to be scientifically meaningful.

## Results

There were no differences between patients and controls on demographic characteristics, but patients differed significantly from controls on depression and anhedonia scales (HAM-D, BDI, and SHAPS) (see Table 1).

### Stage 1: Initial instrumental training

**Figure 2:**
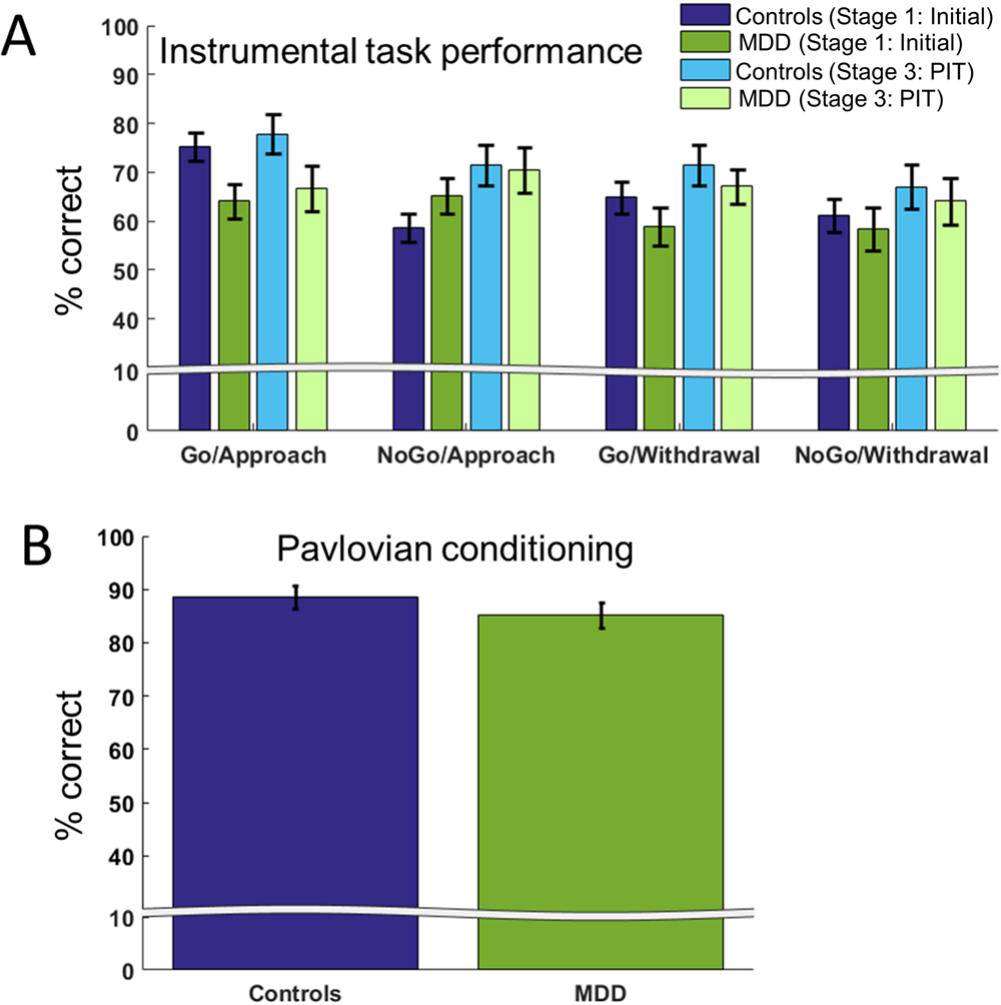
Performance across task stages. (**A**) No significant effect of group on instrumental task performance was detected in Stage 1 (initial instrumental stage) or Stage 3 (PIT stage). Additionally, no significant interaction with group on performance between the stages was observed. (**B)** No group differences were found in the strength of Pavlovian conditioning during Stage 2. Error bars show standard errors of the mean.

Participants demonstrated clear acquisition of the instrumental associations in the instrumental stage (Fig. 2A, Stage 1 - dark bars). Participants performed significantly better in approach than withdrawal blocks (main effect of condition: *F*(1,52) = 5.720, *p = *0.020), with no main effect of action (*F*(1,52) = 2.849, *p* = 0.097) and no condition-by-action interaction (*F*(1,52) = 1.105, *p* = 0.318). There was no evidence that the groups differed in their acquisition of the contingencies in the initial instrumental training stage: all interactions with group were non-significant (group-by-condition: *F*(1,52) = 0.264, *p* = 0.609; group-by-action: *F*(1,52) = 3.142, *p* = 0.082; group-by-condition-by-action: *F*(1,52) = 1.805, *p* = 0.185), and there was no main effect of group (*F*(1,52) = 2.340, *p* = 0.132).

### Stage 2: Pavlovian conditioning

Both groups performed accurately on the Pavlovian choice trials in Stage 2, demonstrating successful encoding of the Pavlovian associations. One patient and one healthy volunteer did not perform substantially above chance accuracy (56%) in this stage, and were therefore excluded from the analyses of Stages 2&3. This left N = 25 patients (mean accuracy = 86%, SD = 11%) and N = 27 healthy volunteers (mean accuracy = 90%, SD = 9%). There was no significant difference in accuracy between the groups (*t*(50) = 1.258, *p* = 0.214; Fig. 2B).

### Stage 3: PIT

Both groups of participants maintained good instrumental task performance during PIT (Fig. 2A, Stage 3 - light bars). There was no main effect of approach/withdrawal condition on accuracy (*F*(1,50) = 1.611, *p* = 0.210). This is in contrast to Stage 1 - participants no longer performed worse in the withdrawal than the approach condition, due to improved performance in the withdrawal condition. We also did not observe a main effect of action (*F*(1,50) = 1.137, *p* = 0.291) or a condition-by-action interaction (*F*(1,50) = 0.309, *p* = 0.581). All interactions with group were non-significant (group-by-condition: *F*(1,52) = 0.167, *p* = 0.685; group-by-action: *F*(1,52) = 1.482, *p* = 0.229; group-by-condition-by-action: *F*(1,52) = 0.849, *p* = 0.361). Again, there was no main effect of group (*F*(1,52) = 1.353, *p* = 0.250).

**Figure 3:**
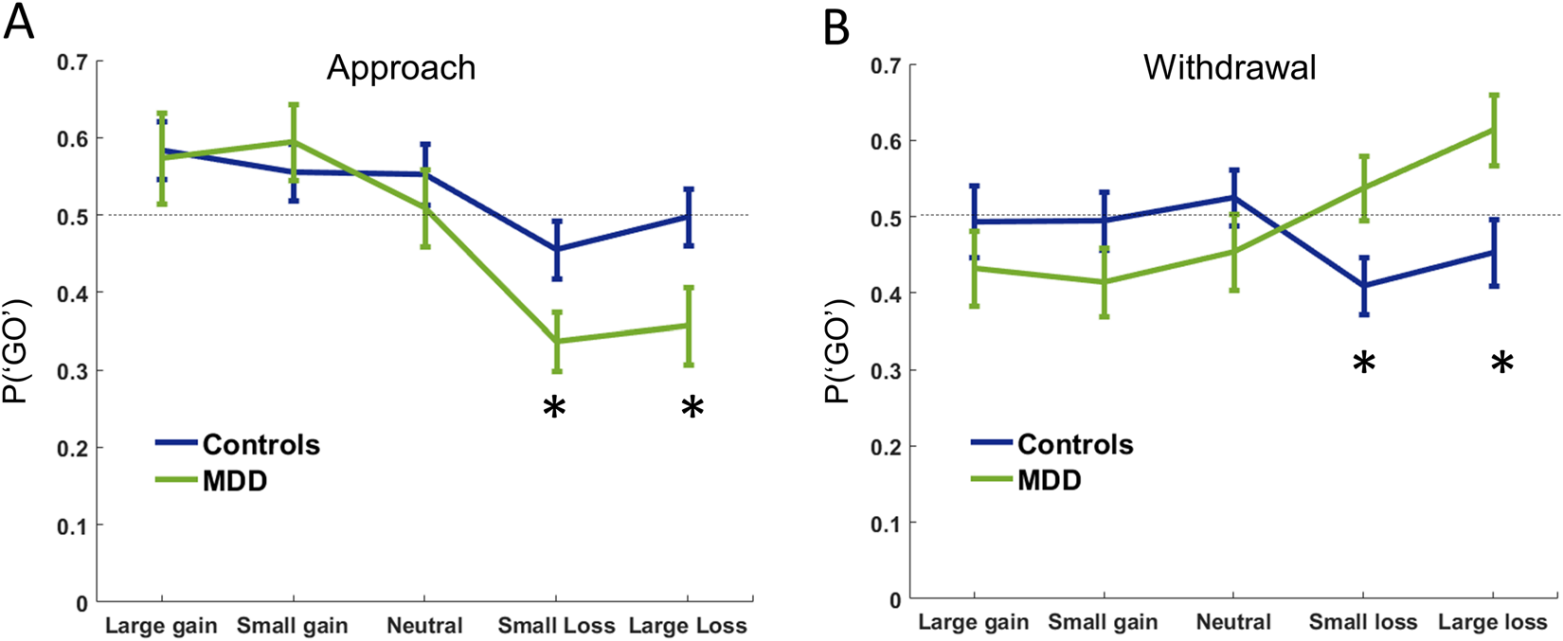
PIT effect. Probability of making a go response (P(‘GO’)) according to each background Pavlovian stimulus valence for approach and withdrawal conditions during the PIT stage. The dotted line indicates a 50% probability of a ‘go’ response, i.e. no response bias. (**A**) In the approach condition, patients with depression made significantly fewer ‘go’ responses on the instrumental task (collected fewer mushrooms) in the presence of Pavlovian background associated with small (−£0.1) and large (−£1) losses. (**B**) In the withdrawal condition, patients made significantly more ‘go’ responses (rejected more mushrooms) in the context of small and large loss-associated background images.

As in previous studies^24^, the PIT effect was examined using the tendency to make a ‘go’ response, regardless of whether this response was appropriate or not. Note that perfect accuracy on the task would yield approximately 50% ‘go’ responses in both approach and withdrawal conditions, equal across all Pavlovian valences. In other words, any deviation in the probability of making a go response from 0.5 (horizontal dotted line in Fig. 3) represents a bias in responding on the task.

We found no significant main effect of approach or withdrawal condition (*F*(4,200) = 0.658, *p* = 0.421), but there was a significant main effect of Pavlovian stimulus valence (*F*(4,200) = 3.427, *p* = 0.01): overall, positive Pavlovian stimuli induced greater rates of ‘go’ responding. There was no valence-by-group interaction (*F*(4,200) = 0.289, *p* = 0.885), and the main effect of group was non-significant (*F*(1,52) = 0.694, *p* = 0.409).

We note that there was also a condition-by-Pavlovian valence interaction (*F*(4,200) = 5.278, *p* < 0.001, η2 = 0.095): overall, Pavlovian stimuli associated with rewards elicited more ‘go’ responses in the approach condition than those associated with losses (Fig. 3A), while the opposite pattern occurred in the withdrawal condition (Fig. 3B) (i.e., a PIT effect). Although this two-way interaction was significant, it is qualified by the three-way interaction below, therefore we do not analyse it further.

The PIT effect was qualified by a significant three-way interaction between group, Pavlovian valence and instrumental condition (*F*(4,200) = 4.511, *p* = 0.002, η^2^ = 0.083). This three-way interaction indicates that the PIT effect was exaggerated in the MDD group (Fig. 3A,B). Linear contrasts showed that this interaction was specifically driven by differences in responses to negative, but not positive, Pavlovian stimuli. In the approach condition, relative to healthy volunteers, MDD patients performed significantly fewer ‘go’ (‘click to collect’) responses in the context of Pavlovian stimuli associated with both small (*t*(1,50) = 2.071, *p* = 0.043) and large (*t*(1,50) = 2.025, *p* = 0.049) losses (Fig. 3A). By contrast, in the withdrawal condition, relative to healthy volunteers, MDD patients performed significantly more ‘go’ (‘click to discard’) responses in the context of Pavlovian stimuli associated with both small (*t*(1,50) = 2.177, *p* = 0.034) and large (*t*(1,50) = 2.553, *p* = 0.014) losses (Fig. 3B). In other words, in MDD patients negative Pavlovian stimuli enhanced responding in the withdrawal condition, but impaired responding in the approach condition. No significant group differences were detected in the context of Pavlovian stimuli associated with large gains, small gains or neutral outcomes, either in the approach or withdrawal conditions. There was no effect of anxiety or previous medication use on the group PIT effect (see Supplemental Information for full analysis of covariates).

To enable direct comparison of our results with previous work (Huys *et al*.^16^), we also fit participants’ responses (P(‘GO’)) to a line, calculating the change in their responses over the five Pavlovian stimuli (i.e., the slope of the PIT effect). This allowed us to examine action specificity: the difference between linear regression coefficients in approach and withdrawal blocks. As all action specificity measures showed a significant departure from Gaussian assumptions (*p* < 0.05, Kolmogorov-Smirnoff test), we employed non-parametric tests (Wilcoxon signed rank and Mann-Whitney U-tests for within- and between-subject comparisons, respectively). We found that in our sample, PIT was action specific in MDD patients (*p* = 0.035, signed rank) but not controls (*p* = 0.876, signed rank). The difference in action specificity between the groups narrowly missed significance (*p* = 0.056), but there were no differences in the approach and withdrawal blocks separately (withdrawal: *p* = 0.179, approach: *p* = 0.369). (See Supplemental Information for a direct comparison of our samples of controls and depressed patients with those of Huys *et al*.).

### Correlations with depressive symptoms

To investigate the impact of depressive symptoms on aversive PIT, we calculated a summary measure describing the aversive PIT effect: the difference between the effect of negative Pavlovian stimuli (relative to neutral stimuli) in the approach block and the effect of negative Pavlovian stimuli (relative to neutral stimuli) in the withdrawal block. We also calculated a summary measure describing the appetitive PIT effect: the difference between the effect of positive Pavlovian stimuli (relative to neutral stimuli) in the approach block and the effect of positive Pavlovian stimuli (relative to neutral stimuli) in the withdrawal block. Note that since the expected PIT effect is reversed from the approach to the withdrawal condition, this summary measure can yield bigger absolute values than for a single condition (e.g., for the aversive PIT in MDD, (PIT_approach_ = −0.15)−(PIT_withdrawal_ = +0.15) = −0.30).

We then conducted correlational analyses between these measures and depression scores. This measure of aversive PIT correlated positively with depressive symptoms overall (both HAM-D and BDI), across both groups (which recapitulates the group effect reported above: HAM-D: *r* = −0.312, *p* = 0.024; BDI: *r* = −0.312, *p* = 0.022), but there was no significant correlation between appetitive PIT and depressive symptoms (HAM-D: *r* = 0.141, *p* = 0.308; BDI: *r* = 0.173, *p* = 0.210). The group effect was also recapitulated in correlations between aversive PIT and anhedonia scores (SHAPS: *r* = −0.378, *p* = 0.005); again, there was no correlation between appetitive PIT and anhedonia (*r* = 0.135, *p* = 0.335). However, depression did not correlate with either measure in the healthy volunteer or the MDD group separately (according to the HAMD, aversive PIT: healthy volunteers *r* = −0.277, *p* = 0.162, MDD patients *r* = 0.136, *p* = 0.515; appetitive PIT: healthy volunteers: *r* = 0.228, *p* = 0.254, MDD patients *r* = −0.027, *p* = 0.515); this was also the case for depression as measured by the BDI, and anhedonia (all *p* > 0.1).

## Discussion

We investigated how negative biases in depression might drive depressive behaviours using a PIT paradigm, which measures the degree to which previously-conditioned cues encourage or inhibit instrumental actions. We hypothesized that depressed patients would show a greater influence of loss-associated Pavlovian cues but a reduced impact of gain-associated cues on both approach and withdrawal behaviour. We found strong support for one aspect of our hypothesis: depression was associated with a greater influence of aversive Pavlovian cues on behaviour, compared to healthy controls. That is, in patients, cues associated with loss decreased the number of approach actions, but increased the number of withdrawal actions. This finding was not driven by any differences in performance on either Pavlovian or instrumental task learning, and was specific to the loss domain. We did not support the second aspect of our hypothesis: we found no strong suggestion of diminished appetitive PIT in depression.

Our findings add to the rich evidence for enhanced processing of negative information in depression, including memories for unpleasant events^30^ and the effect of negative feedback on behaviour^18,19^. Our PIT data extend this prior work, presenting a potential mechanism that could explain the influence of previous negative events on behaviour in depression: namely, that environments and stimuli associated with adverse events could exert an exaggerated and maladaptive influence on depressed patients’ behaviour. This provides a link between aberrant reward and punishment processing observed experimentally and symptoms commonly reported in depression. Previous adverse outcomes might cause a patient to excessively avoid any similar situations, leading to motivational deficits and social withdrawal.

Interestingly, the effect cannot be explained by simple action inhibition in the context of negatively-associated cues, since action was both invigorated (in the withdrawal blocks) and suppressed (in the approach blocks) by the same negative cues. The influence of cues on behaviour through PIT provides a plausible mechanism for the transfer of implicit biases in depression onto thoughts and behaviours.

In general, an exaggerated influence of past negative experiences or outcomes on current behaviour in depression could encourage both active and passive avoidance symptoms in depression (such as social withdrawal or anhedonia, a loss of pleasure derived from normally-pleasurable activities). Recent research has identified a key role for subcortical structures such as the habenula in aversive Pavlovian conditioning and passive avoidance behaviours in healthy volunteers^31^. Additionally, habenula function has been shown to be disrupted in MDD^32,33^, and smaller habenula volumes are associated with anhedonia. Therefore, future studies combining PIT and functional neuroimaging of the habenula in MDD may provide new insights in the cognitive and neural mechanisms underlying the motivational symptoms of the disorder.

Pavlovian cues may also influence cognition in depression by promoting negative ideation. However, these behavioural and cognitive patterns may not be specific to depression. Instead, it is likely that an exaggerated influence of negative Pavlovian processing contributes to depressive cognition and behaviour across several different psychiatric disorders, potentially driving similar withdrawal in social anxiety disorder, or excessive avoidance of panic-related environments, in the case of panic disorder. It would be informative to investigate PIT in these different contexts in future studies.

### Pharmacology of the PIT effect

The action- and valence-specificity of our results may be underpinned by interactions between dopaminergic and serotonergic systems. Studies in animals have primarily implicated the dopamine system in PIT: phasic dopamine spikes are temporally coupled to Pavlovian cue onset, and predict instrumental lever-pressing in rats^34^, while inactivation of the ventral tegmental area, a dopamine-rich region, diminished appetitive PIT^13^. However, the majority of the animal PIT literature has focused on reward-predictive Pavlovian cues in the context of appetitive PIT.

By contrast, many studies support the involvement of the serotonin system in aversive processing^21^, though rarely has this been explored with PIT. In one study, depletion of tryptophan (a precursor of serotonin) dampened both Pavlovian and instrumental aversive behaviours^35^, suggesting that the influence of aversive Pavlovian cues on behaviour may be driven by serotonin neurotransmission. In contrast, there is also evidence that tryptophan depletion *enhances* the motivational influence of aversive stimuli on instrumental behaviour without affecting appetitive cues (while depletion of dopamine precursor tyrosine reduces appetitive PIT)^36^. Although both studies suggest the involvement of serotonin in aversive processing, their conflicting results underline the need to clarify the role of serotonin in PIT. Indeed, the interaction between the effect of depression on PIT and the separate effects of monoamine pharmacology on PIT may explain the discrepancy between our report and the only other investigation of PIT in sample of patients with depression, which included medicated patients^16^.

To better compare our results, we also conducted analyses of action specificity (using the calculation described in Huys *et al*.^16^). We found here that PIT was action specific in depressed patients but not controls (and the difference between the groups narrowly missed significance); in contrast, Huys *et al*.^16^ found PIT was action specific in controls but not depressed patients. In both studies, there was a marginal group difference in action-specificity (though in opposite directions in the two studies). Interestingly, Huys *et al*.^16^ also found action specificity in currently-depressed patients who recovered 4–6 months later, which was driven by effects in the withdrawal condition. It is possible that our patient sample more closely resembled the subsample of patients in the previous study who showed action-specificity. This may reflect heterogeneity in the cognitive profile of depressed patients. Moreover, as PIT was not action-specific in our sample of healthy controls, it may also imply that PIT effects are variable across healthy populations. The source of this variability is unclear and would be better addressed by experiments in larger samples, which could examine whether action specificity is associated with demographic or other cognitive measures.

This precise version of the task has been used in two previous studies^16,24^, which included an overlapping sample of healthy controls. The strong action-specificity reported in the control group in those studies was not apparent here, and this seems related both to a lesser PIT effect during approach, and possibly a reversal of the effect direction during withdrawal. Overall, the literature suggests relatively stable PIT effects on approach behaviour^37–39^, but somewhat weaker and more variable effects on withdrawal^14,15^. This general pattern is consistent across the studies, though the precise reasons for this have as yet to be elucidated. In addition, though differences in the effect of Pavlovian stimuli on approach and withdrawal behaviour have a strong conceptual basis, PIT paradigms examine modulations of ongoing behaviour by acquired Pavlovian expectations, and as such tend to yield relatively small effects. Interactions, such as the action-specificity, between different PIT effects are by nature even smaller, and should hence be interpreted cautiously.

Of note, there were also differences in patient samples between the studies: first, the previous study sample excluded patients with comorbid anxiety; second, our sample excluded patients taking psychotropic medication. With respect to the two differences between our studies, including anxiety diagnosis as a covariate in our model did not produce a significant interaction with any main or interaction effects (see Supplemental Information), nor did including patients with anxiety in the previous study weaken their reported group effect^16^. Therefore, it is more likely that medication status played a larger role in the differences between the two studies.

While some tryptophan depletion findings might indicate enhanced aversive PIT in depression^36^, where disruptions to serotonin transmission have long been implicated^40^, others would predict the exact opposite: an attenuated aversive PIT^15^. Of course, serotonin deficits are not thought to fully explain depressive symptoms^41^. Nonetheless, the intriguing possibility remains that while animal literature has strongly linked phasic dopamine release to appetitive PIT^34^, the serotonin system may be implicated in aversive PIT, linking disorders of serotonin dysfunction to aberrant aversive PIT, and modulation of this aversive PIT effect as a possible result of serotonergic medication.

### Limitations

Some limitations of this study merit comment. First, we must be cautious interpreting these results as specific to MDD. Fifteen patients (58% of our MDD sample) met criteria for generalized anxiety disorder (GAD), which could have augmented the difference in aversive PIT effect between groups. However, the two disorders are closely related, with some arguing that GAD ought not be viewed as independent from MDD^42^. Additionally, while our sample size was sufficient to detect a main effect of large size (d = 0.8^43^), it was insufficiently powered to detect smaller effects. Most importantly, for the within-group correlation analyses (N = 27 and 25 for healthy controls and MDD, respectively), we had only 71% power to detect a medium effect size of r = 0.45 – this makes the lack of significant correlations with symptoms difficult to interpret. Similarly, our study was designed to detect a main effect of group, but not the effects of possible covariates, for example the contribution of demographic variables to the PIT effect, such as socioeconomic status, which is known to alter cognitive processing^44^. Our results need to be confirmed in future, larger studies, ideally with enough subjects to examine effects of mood and anxiety separately and adequate power to examine possible moderators of the effect. This would allow us to account for clinical differences, but also to detect smaller effects, and to test for correlations between behaviour and symptoms with greater confidence.

## Conclusions and Future Directions

We identified an exaggerated influence of aversive Pavlovian stimuli on instrumental choice, dampening instrumental approach but enhancing instrumental withdrawal behaviour. This finding is consistent with the broader literature indicating exaggerated negative information processing in depression, and offers a plausible explanation for the automatic impact of negative biases on decision-making in depression. However, its relationship to previous PIT findings is less clear, with inconsistent observations across dietary tryptophan depletion studies^15,36^, and a medicated depressed sample^16^. In future it would be interesting to characterize the nature of disrupted PIT in depression more comprehensively, investigating whether at-risk and remitted samples show intact or abnormal PIT, and to illuminate the effects of pharmacological and psychological therapies on PIT.

## Electronic supplementary material

Supplementary Information

## Data Availability

The datasets generated during and/or analysed during the current study are available in the Open Science Framework repository, osf.io/3p8gr. The dataset associated with this study is available from the corresponding author on reasonable request and in accordance with local ethics rules.
