# Supplementary material for: Depression is associated with enhanced aversive Pavlovian control over instrumental behaviour
Source: Sci Rep. 2018 Aug 22;8:12582. doi: 10.1038/s41598-018-30828-5 (PMC6105578; doi:10.1038/s41598-018-30828-5)
Supplement: Supplementary file 1 — Supplementary Information [file 41598_2018_30828_MOESM1_ESM.docx]

Supplemental information

**Depression is associated with enhanced aversive Pavlovian control over instrumental behaviour**

Nord, C.L.^†^ , Lawson, R.P. ^†^, Huys, Q.J.M., Pilling, S., Roiser, J.P.

^†^These authors contributed equally.

*Effect of Stage 1 and Stage 2 performance*

Group differences at Stage 3 remained significant when covarying for baseline performance in Stage 1 (Instrumental), for all conditions. The three-way interaction between group, condition, and valence was significant when controlling for “go” performance in the approach block (F(4,196)=4.125, p=0.003), “no go” performance in the approach block (F(4,196)=5.103, p=0.001), “go” performance in the withdrawal block (F(4,196)=3.651, p=0.007), and “no go” performance in the withdrawal block (F(4,196)=4.215, p=0.003).

Group differences at Stage 3 also remained significant when covarying for baseline conditioning in Stage 2 (Pavlovian): F(4,196)=4.327, p=0.002).

Finally, we tested whether there was an interaction between baseline Pavlovian conditioning and the PIT effect across all participants. This was non-significant (F(4,196)=9.647, p=0.630).

*Analysis of anxiety (GAD) diagnosis*

We repeated our analysis of the third (PIT) stage of the task including anxiety comorbidity as a covariate. We classified patients according to criteria for Generalized Anxiety Disorder (GAD) on the MINI Neuropsychiatric Interview. Sixteen patients (59% of our sample) met criteria for GAD. We included GAD diagnosis as a between-subject factor in a repeated-measures analysis of variance conducted only on the patient group.

We found that diagnosis of GAD did not interact with the main effect of approach or withdrawal, *F*(1,23)=0.359, *p*=0.555, the main effect of Pavlovian stimulus valence, *F*(4,92)=0.478, *p*=0.752, or the interaction between approach or withdrawal with Pavlovian stimulus valence (i.e., the PIT effect), *F*(4,92)=0.842, *p*=0.502.

*Analysis of previous medication use*

We repeated our analysis of the third (PIT) stage of the task including previous medication use as a covariate, finding that there was no interaction between previous medication use at the effect of group on the PIT effect (*F*(4,96)=0.806, *p*=0.524); inclusion of this variable as a covariate strengthened the three-way interaction between group, instrumental condition, and Pavlovian valence (*F*(4,96)=7.147, *p*<0.001).

*Comparison with Huys et al., 2016*

We compared the action specificity measure (slope of approach line – slope of withdrawal line) between the groups include in the present study and the groups included in Huys et al. (2016). We found that the present sample of healthy controls differed from those in Huys et al. (2016) (*t*(65)=2.05, *p*=0.044), and that the samples of MDD patients also differed (*t*(26.87)=2.62, *p*=0.014). See figure below:


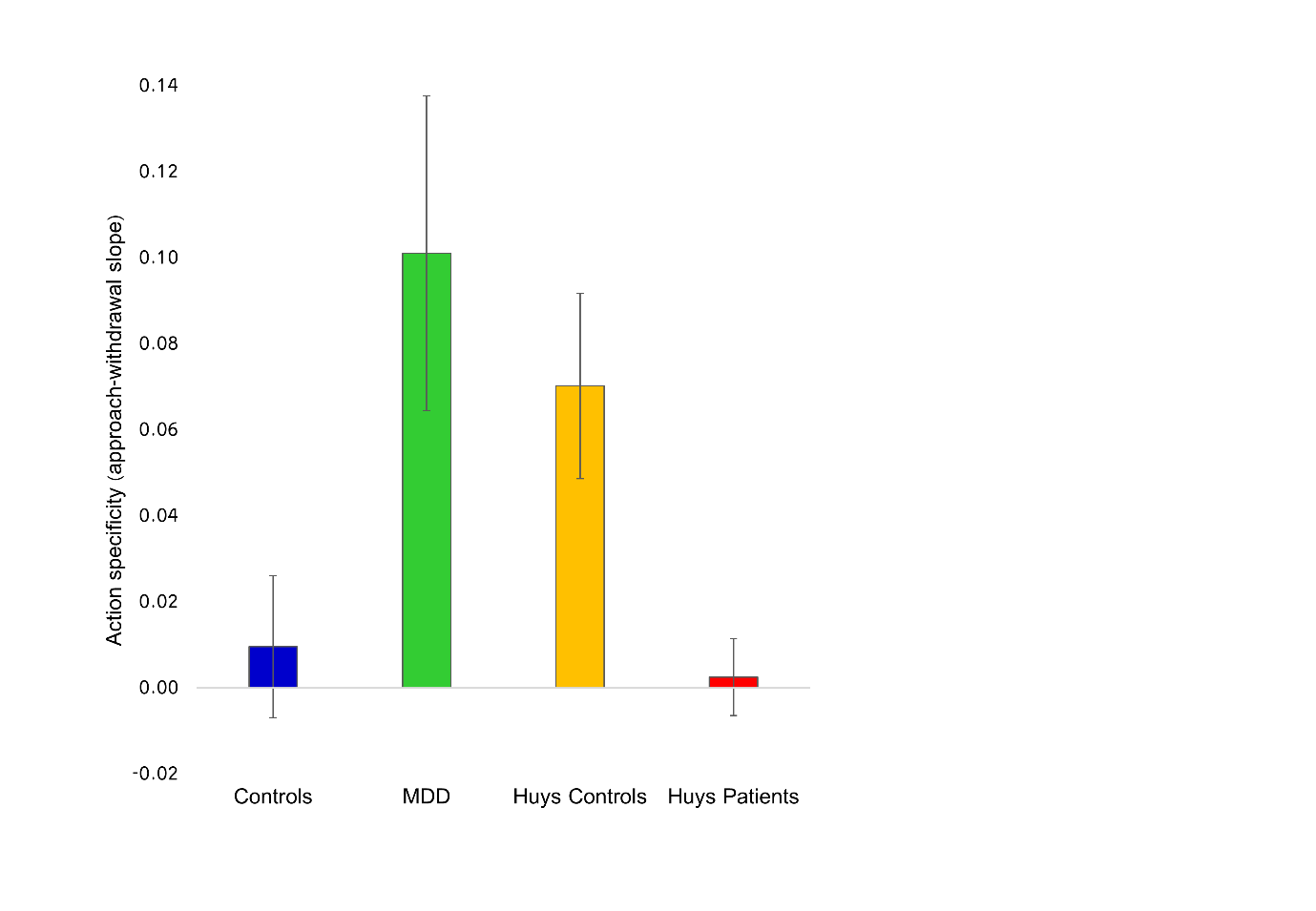


**Supplemental Figure 1.** Action specificity (mean approach – mean withdrawal slope) for the present sample of controls and MDD patients, contrasted with the previous (Huys et al., 2016) sample of controls and MDD patients.
